# Association between the Density of Physicians and Suicide Rates in Japan: Nationwide Ecological Study Using a Spatial Bayesian Model

**DOI:** 10.1371/journal.pone.0148288

**Published:** 2016-02-03

**Authors:** Hideaki Kawaguchi, Soichi Koike

**Affiliations:** 1 Department of Medical Informatics and Economics, The University of Tokyo, Tokyo, Japan; 2 Division of Health Policy and Management, Center for Community Medicine, Jichi Medical University, Tochigi, Japan; 3 Department of Health Management and Policy, Graduate School of Medicine, The University of Tokyo, Tokyo, Japan; Institute for Health & the Environment, UNITED STATES

## Abstract

**Background:**

Regional disparity in suicide rates is a serious problem worldwide. One possible cause is unequal distribution of the health workforce, especially psychiatrists. Research about the association between regional physician numbers and suicide rates is therefore important but studies are rare. The objective of this study was to evaluate the association between physician numbers and suicide rates in Japan, by municipality.

**Methods:**

The study included all the municipalities in Japan (n = 1,896). We estimated smoothed standardized mortality ratios of suicide rates for each municipality and evaluated the association between health workforce and suicide rates using a hierarchical Bayesian model accounting for spatially correlated random effects, a conditional autoregressive model. We assumed a Poisson distribution for the observed number of suicides and set the expected number of suicides as the offset variable. The explanatory variables were numbers of physicians, a binary variable for the presence of psychiatrists, and social covariates.

**Results:**

After adjustment for socioeconomic factors, suicide rates in municipalities that had at least one psychiatrist were lower than those in the other municipalities. There was, however, a positive and statistically significant association between the number of physicians and suicide rates.

**Conclusions:**

Suicide rates in municipalities that had at least one psychiatrist were lower than those in other municipalities, but the number of physicians was positively and significantly related with suicide rates. To improve the regional disparity in suicide rates, the government should encourage psychiatrists to participate in community-based suicide prevention programs and to settle in municipalities that currently have no psychiatrists. The government and other stakeholders should also construct better networks between psychiatrists and non-psychiatrists to support sharing of information for suicide prevention.

## Introduction

Suicide is a serious public health problem. According to the World Health Organization, about 800,000 people die by suicide every year worldwide and suicide was the 15^th^ most common cause of death in 2012 [1]. The suicide rate in Japan is high, over 15 per 100,000 people [1].

There is considerable regional variation in suicide rates. Suicide rates are often higher in rural areas than urban, a phenomenon that has been observed in, for example, China [2], the United States [3], Australia [4], Finland [5] and the United Kingdom [6]. Regional variation in suicide rates has also been observed in Japan; for example, the suicide rate in Akita prefecture is higher than in other areas, and this regional disparity is gradually becoming more pronounced [7].

The regional disparity in suicide rates has a complicated mechanism and many contributory factors have been identified. These include some socioeconomic factors, for example, unemployment rate [8], population density [9], and average per capita income [10]. One important factor may be the unequal distribution of the healthcare workforce. In a study in Hungary, a negative correlation between the density of physicians and regional suicide rates was shown, but this study did not consider socioeconomic confounders [11]. A study at the county level in the United States found similar results [12]. It is also important to conduct studies across smaller areas because the size of the area might affect results about the relationship between socioeconomic factors and suicide rates [13]. A small-area study in Finland showed that the prominence of outpatient services was associated with low suicide rates after adjustment for socioeconomic factors [5]. In a nationwide small-area study in Austria, density of neither psychiatrists nor general practitioners was statistically associated with regional suicide rates [14].

Research into the association between regional mental health workforce and suicide rates is important to formulate policies for preventing suicide, but not many studies have focused on this. Nationwide and municipality-level studies are particularly rare. The aim of this study was to evaluate the association between density of physicians and suicide rates by municipality.

## Materials and Methods

### Study design

This was an observational cross-sectional study using publicly-available secondary data. Japan consists of 47 prefectures, of which the smallest administrative division is municipalities. We targeted all the municipalities in Japan as at January 11, 2015 (n = 1,896).

### Data sources

Socioeconomic data were obtained from the national Japanese government database, e-Stat, which holds various data at municipality level, including socioeconomic and sanitary data [15]. Geographical information, such as boundary data for the municipalities, was obtained from data published by ESRI Japan Inc. [16]. Suicide data were obtained from the official mortality database for suicides by municipality published by the Japanese Cabinet Office [17].

The Japan Ministry of Health, Labour and Welfare (MHLW) conducts a detailed biennial investigation into the working conditions of physicians, dentists and pharmacists. Details from this investigation supplied numbers of physicians and psychiatrists in each municipality [18].

As socioeconomic covariates, we used all available socioeconomic factors from e-Stat that had been identified in previous studies [5, 13–14]. These were: population density (people per hectare), average per capita income (in 10,000 yen), unemployment rate (%), proportion of university graduates per population over 15 years old (%), crime rate (incidence per 1,000 population), and divorce rate (incidence per 1,000 population). Data for all socioeconomic factors except for the crime rate were from fiscal year 2010, and crime data were from fiscal year 2009. Data for physicians and suicide rates were from fiscal year 2010. To control for municipal mergers since 2010 and give figures for 2015 municipalities, we amalgamated data from 2010. To manage municipal amalgamation, we summed data for the municipalities amalgamated in 2009 and 2010, 12 in total, creating six new municipalities. The cities of Sagamihara and Kumamoto were designated by government ordinance and divided into three and five districts, respectively. We used the average data for each city in 2009 and 2010 to provide data for each district in 2015.

### Statistical analysis

We calculated ratios for mortality from suicide based on standard mortality ratios (SMRs). SMR has commonly been used to adjust for age and sex in previous studies about suicide rates and we used this index because of its high comparability [8, 14]. SMR is calculated by dividing the observed number by expected number of suicides. We calculated ratios separately for men and women, and for eight age subgroups: under 20, 21–30, 31–40, 41–50, 51–60, 61–70, 71–80, and over 80 years. We calculated national suicide rates in each subgroup, then multiplied the national suicide rates by population of each subgroup in the municipality to obtain the expected number of suicides.

Because municipalities can vary in size, SMR might be affected by larger variances in small municipalities than in large ones. To adjust for this problem, we estimated smoothed SMR (sSMR) using a hierarchical Bayesian model. A conditional autoregressive model (CAR) is commonly used to estimate sSMR considering neighborhood structure [14, 19–21]. We chose the Leroux CAR model. Lee used simulations to establish that this was consistently suitable for various spatial correlation scenarios [22]. We assumed a Poisson distribution for the observed number of suicides and set the expected number of suicides as the offset variable. We used Markov chain Monte Carlo (MCMC) simulations with 110,000 iterations and 10,000 burn-in. Typically, there is likely to be a correlation with spatial data relating to locations, or spatial autocorrelation [23]. We calculated Moran’s I for sSMR and checked whether sSMR had any spatial autocorrelation [23].

To investigate the association between health workforce and suicide rates, we included the number of physicians per 1,000 population, a binary variable for the presence of psychiatrists, and socioeconomic factors in the Leroux model as explanatory variables. Because many municipalities have no psychiatrists (n = 852), we set the dummy binary variable as 1 if a municipality had at least one psychiatrist and 0 if there were no psychiatrists. We evaluated multicollinearity of socioeconomic covariates using the variance inflation factor (VIF). VIF is calculated for each variable using a regression of the variable on all the other variables [24]. Allison suggested the need for concern when a VIF is greater than 2.5 [24]. Average per capita income (VIF = 3.34) and proportion of university graduates in the population over 15 years old (VIF = 3.36) both showed this characteristic, and the variables strongly correlated (r = 0.829). We removed the proportion of university graduates because it had the greatest VIF and put all the other variables into the Leroux model. We estimated the relative risks (RR) and 95% Bayesian credible intervals (CIs) of each variable.

All analyses were conducted using R v.3.1.2 [25].

### Ethics

This study used only publicly available secondary data and approval from an ethics review board was not necessary, according to the *Ethical Guidelines for Medical and Health Research Involving Human Subjects in Japan* [26].

## Results

**Fig 1:**
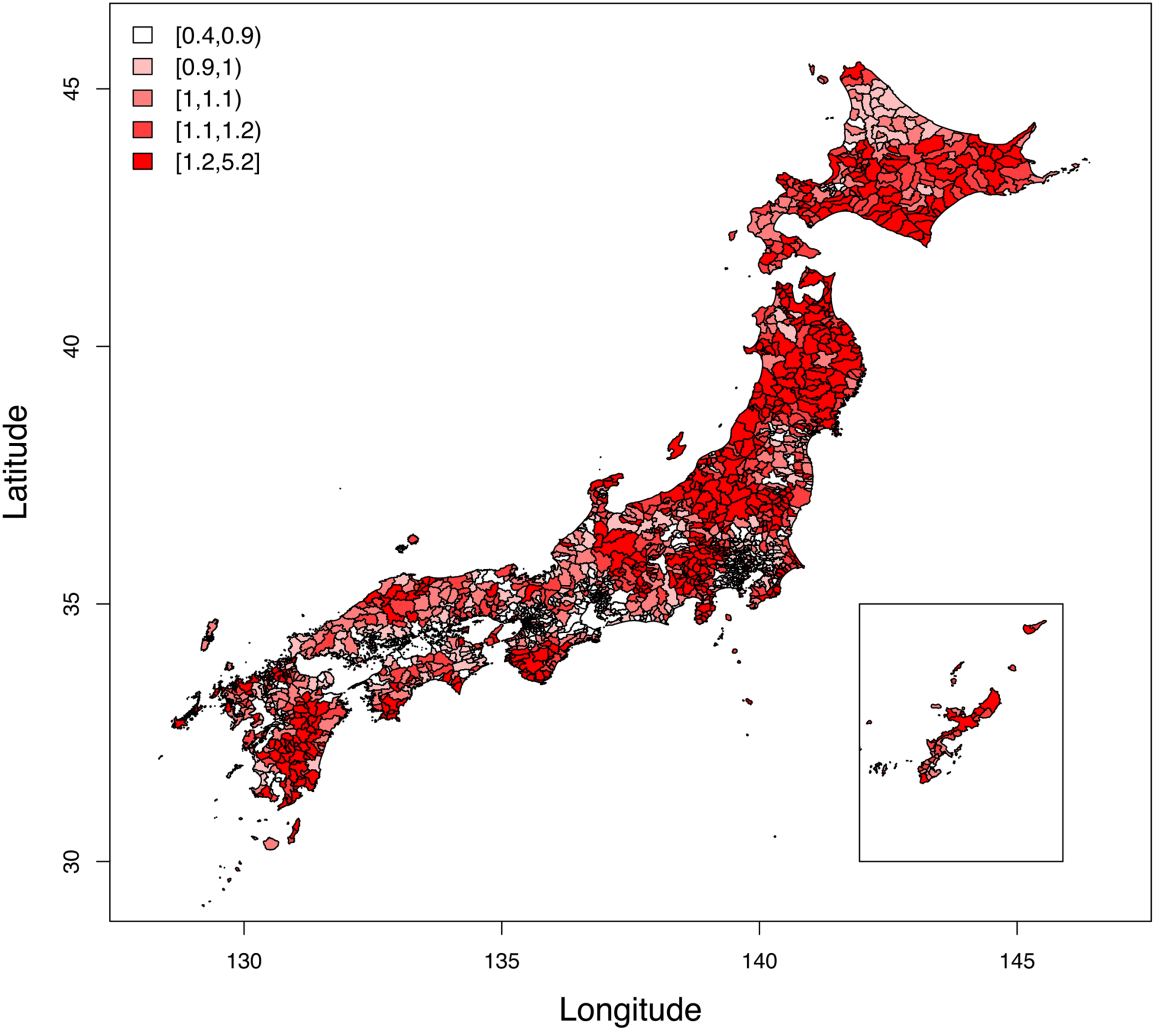
smoothed standardized mortality ratio for suicide in Japan, 2010.

The mean and standard deviation of sSMR were 1.096 and 0.247, indicating a range across municipalities. Fig 1 shows sSMR of suicide rates in Japan in 2010 and shows the variety across municipalities. The sSMR in big cities, such as Tokyo and Osaka, were smaller than other areas, which suggested an autocorrelation. Moran’s I statistic for sSMR was 0.597 (p < 0.001), which showed the strong autocorrelation of sSMR in Japan.

Table 1 shows descriptive data for the 1,896 municipalities included in this study. The number of physicians varied quite widely and 26 municipalities had no physicians. Almost half of the municipalities (852) had no psychiatrists.

**Table 1:** Characteristics of 1,896 municipalities in Japan.

		Interquartile range	
Characteristics	Median	1st	3rd
**sSMR (2010)**	**1.080**	**0.942**	**1.216**
**Physicians per 1,000 population (2010)**	**1.578**	**0.716**	**1.832**
**Unemployment rate (%) (2010)**	**6.150**	**5.118**	**7.315**
**Average per capita income in 10,000 yen (2010)**	**271.7**	**248.7**	**305.9**
**Population density per hectare (2010)**	**600.5**	**295.3**	**1703.4**
**Crime rate per 1000 population (incidence per 1,000 population) (2009)**	**10.89**	**6.920**	**16.19**
**Divorce rate (incidence per 1,000 population) (2010)**	**1.789**	**1.452**	**2.105**
**Proportion of university graduates (%) (2010)**	**10.80**	**7.700**	**15.53**

sSMR = smoothed standardized mortality ratio

Table 2 shows the detailed results of the CAR Leroux model. The absolute values of the Geweke diagnostic for each parameter were all less than 1.96, which showed that all the parameters used in MCMC converged [27]. Table 2 shows that the association between the density of physicians and sSMR was significant (RR = 1.015, CI = 1.004–1.026). Across Japan, an increase of 130,000 physicians, or about 45% of the total, increases suicide rates at a rate of 1.5%. Suicide rates in municipalities with at least one psychiatrist were significantly lower than in other municipalities (RR = 0.9, CI = 0.852–0.947). Unemployment rate and crime rate per 1,000 population were positively associated with suicide rates, while average per capita income in 10,000 yen, population density per hectare and divorce rate were negatively associated.

**Table 2:** Results of estimating the Leroux model for suicide mortality ratio.

		Credible interval		
	Median	2.5%	97.5%	Geweke diagnostic
**Intercept**	**0.539**	**0.344**	**0.735**	**−0.8**
**Physicians per 1,000 population**	**0.015**	**0.004**	**0.026**	**−1.2**
**Presence of psychiatrists**	**−0.100**	**−0.148**	**−0.053**	**−0.8**
**Unemployment rate**	**0.017**	**0.004**	**0.030**	**1.4**
**Average per capita income**	**−0.0014**	**−0.002**	**−0.0008**	**1.4**
**Population density**	**−0.002**	**−0.003**	**−0.001**	**−0.1**
**Crime rate per 1,000 population**	**0.006**	**0.003**	**0.008**	**1.5**
**Divorce rate**	**−0.085**	**−0.139**	**−0.032**	**−1.6**

## Discussion

Regional disparity in suicide rate is a very serious problem and it is important to know whether there is an association between health workforce and regional suicide rates, so that effective policies can be developed to reduce these disparities. We found two major results with regard to the relationship between physician numbers and suicide rates.

First, sSMRs of municipalities with at least one psychiatrist were lower than those of other municipalities. This result shows the importance of having psychiatrists in each municipality. It is inconsistent with a previous study in Austria, but agrees with a study in the United States [12, 14]. The Austrian study showed that the density of psychiatrists in the United States (0.14 per 1,000) was higher than in Austria (0.02 per 1,000) and that the effect of psychiatrists on suicide rates had been shown in the United States [14]. The density of psychiatrists in Japan (0.11 per 1,000) was also much higher than in Austria, and might play a key role in suicide rates [14]. It might be important to encourage psychiatrists to settle in municipalities that currently have no psychiatrists.

Because there are limited numbers of psychiatrists, we might also need to increase the quality of interventions. The MHLW has set up a project team for the management of depression and prevention of suicide. Community-based approaches to suicide prevention by municipalities have been promoted [28], and some have been successful. For example, Ono and colleagues showed that community-based interventions were effective in reducing suicide rates among men and older people in rural areas [29].

In their study, non-medical ‘gatekeepers’ were used to identify people who might commit suicide at an early stage. The effect of the intervention program could potentially be increased by using psychiatrists to educate and support gatekeepers. To improve the quality of such interventions, it will be important to raise awareness of such interventions among psychiatrists and to encourage them to participate. It might, however, be difficult to impose duties on psychiatrists, because many of them are employed by hospitals. The government should therefore work on persuading not only psychiatrists but also hospitals to participate in these initiatives.

Second, our findings showed that there was a positive relationship between the number of physicians and suicide rates. Considering the negative relationship between the presence of psychiatrists and suicide rates, this result suggests increasing the number of physicians who are not psychiatrists (hereafter called “non-psychiatrists”) does not improve suicide rates. This might be because of differences in methods of assessment and treatment of psychiatric patients, and particularly in the prescription of psychotropic drugs and assessment of suicide risk.

A study in Japan suggested that an increase in overdose suicide in younger generations after 2000 was the result of suboptimal prescription of psychotropic drugs [30]. Non-psychiatrists, who make up the majority of physicians in Japan, generally have less knowledge of psychotropic drugs than psychiatrists, but often prescribe them. If there are more non-psychiatrists in a municipality, there will be more opportunities for suboptimal prescription of psychotropic drugs. The association between suicide rates and the density of physicians in this study might be because of an association between rates of suboptimal prescription and suicides. Our result was, however, different from previous studies in other countries [5, 11, 14]. This difference might result from higher rates of suboptimal prescription of psychotropic drugs in Japan. A previous study suggested that consumption levels of benzodiazepine agents in Japan tended to be comparatively high and that this high consumption might reflect suboptimal prescription and associated abuse [30]. Future studies should examine this possibility more closely.

A study in the United Kingdom pointed out that it was difficult for general practitioners to assess suicide risk in primary care, partly because of a relative lack of suicide risk assessment training [31]. Non-psychiatrists in Japan are similar to general practitioners in the sense that they have little training in suicide risk assessment.

To support non-psychiatrists in developing their ability to assess psychiatric patients, it might help to create networks of psychiatrists and other physicians to share information about suicide prevention. This will increase opportunities for psychiatrists to provide information to and share skills with non-psychiatrists. Evidence shows that general practitioners often suffer from an environment that makes it hard to consult psychiatrists [32]. This phenomenon is common in Japan and there are barriers between non-psychiatrists and psychiatrists. Community-based interventions for suicide prevention demand cooperation between non-psychiatrists and psychiatrists, and the MHLW has provided support in this regard [28].

To construct better networks between physicians, it may be important to establish a clear qualifications system such as a specialist system. Without such a system, it is hard to clarify the roles of physicians in networks, and network infrastructure may not work as well. After resolving these problems, the government should provide more opportunities for psychiatrists to share their expertise in psychiatric examination with non-psychiatrists, which might close the gap.

Our results for all socioeconomic variables except for divorce rate coincided with previous studies in various countries [8–10]. A positive relationship between divorce and suicide has been found in Western countries in particular, although this relationship tended to vary across Eastern Asian countries [33]. Stack found no association between divorce and suicide in Japan [34]. In another Japanese study, a negative association between suicide rates of older people and divorce rates was found in an area of Osaka prefecture [35]. Motohashi pointed out that the relationship between divorce and suicide rate depended on the year studied [36]. Further studies of social conditions at various times are necessary.

### Limitations

This study had several limitations. First, it was a municipality-level ecological analysis and did not account for patient-level factors that might contribute to suicide, such as mental disorders, drug misuse, and other personal factors. Further studies using individual data are necessary to overcome this problem. Second, it was a cross-sectional study and this design does not permit us to estimate any causal relationships, only associations. In future, longitudinal studies will be necessary. Third, the study considered only spatial autocorrelation and not spatial heterogeneity. For example, a statistical model might vary in each region. In future, other spatial statistical models should be used to address this problem. Fourth, we could only obtain limited socioeconomic variables at municipality level from the national database, e-Stat. Further studies using other databases are necessary to overcome this.

Despite these limitations, this study provides the first evidence from Japan that we are aware of that shows an association between health workforce and regional suicide rates. Few studies have assessed this association and our findings therefore help to fill a gap in the literature. We have enhanced the study’s accuracy by using spatial statistical models to consider spatial autocorrelation.

It will be important for future research to assess regional differences in associations between health workforce and suicide rates by analyzing spatially varying relationships. This will enable more detailed policy recommendations for distribution of the health workforce to improve suicide prevention.

## Conclusions

To reduce the regional disparity in suicide rates, the government should encourage psychiatrists to participate in community-based programs for suicide prevention and to settle in municipalities that currently have no psychiatrists.

The government should also construct networks for sharing clinical information about patients at high risk of suicide, and thus reduce professional isolation of non-psychiatrists. To do so, it might be helpful to establish a clear qualifications system such as a specialist system.
